# Single‐nucleus transcriptome analysis reveals disease‐ and regeneration‐associated endothelial cells in white matter vascular dementia

**DOI:** 10.1111/jcmm.17315

**Published:** 2022-05-11

**Authors:** Daniel N. Mitroi, Min Tian, Riki Kawaguchi, William E. Lowry, S. Thomas Carmichael

**Affiliations:** ^1^ Department of Psychiatry and Biobehavioral Sciences David Geffen School of Medicine at UCLA Los Angeles California USA; ^2^ Department of Molecular, Cell and Developmental Biology UCLA Los Angeles California USA

**Keywords:** angiogenesis, disease‐associated endothelial cells, snRNA‐seq, vascular dementia, white matter

## Abstract

**Background:**

Vascular dementia (VaD) is the accumulation of vascular lesions in the subcortical white matter of the brain. These lesions progress and there is no direct medical therapy.

**Aims:**

To determine the specific cellular responses in VaD so as to provide molecular targets for therapeutic development.

**Materials and Methods:**

Single‐nucleus transcriptome analysis was performed in human periventricular white matter (PVWM) samples of VaD and normal control (NC) subjects.

**Results:**

Differential analysis shows that cell type‐specific transcriptomic changes in VaD are associated with the disruption of specific biological processes, including angiogenesis, immune activation, axonal injury and myelination. Each cell type in the neurovascular unit within white matter has a specific alteration in gene expression in VaD. In a central cell type for this disease, subcluster analysis of endothelial cells (EC) indicates that VaD contains a disease‐associated EC subcluster that expresses genes associated with programmed cell death and a response to protein folding. Two other subpopulations of EC in VaD express molecular systems associated with regenerative processes in angiogenesis, and in axonal sprouting and oligodendrocyte progenitor cell maturation.

**Conclusion:**

This comprehensive molecular profiling of brain samples from patients with VaD reveals previously unknown molecular changes in cells of the neurovascular niche, and an attempt at regeneration in injured white matter.

## 1 INTRODUCTION

Vascular dementia is one of the most common causes of dementia, accounting for ~15%–20% of cases, and co‐exists with Alzheimer's Disease in the most prevalent form of dementia, ‘mixed dementia’.^1^, ^2^ Vascular dementia occurs as ischaemic lesions in the subcortical white matter of the brain. These lesions progress radially over time, causing myelin loss and axonal abnormalities.^3^, ^4^ Unlike the other major white matter disease in adults, multiple sclerosis, there is no process of repair in the ischaemic white matter lesions in vascular dementia.^1^, ^5^ The cellular biology of the white matter disease progression and repair is poorly defined, in part because animal models do not replicate all aspects of the human disease.

The disruption of the molecular pathways of specific cell types can contribute to progression of the disease or in a possible repair of the injured white matter. Recent studies have utilized microarray or direct bulk RNA sequencing analyses in cerebellum or cortex to quantify differences in gene expression patterns and highly connected networks among individuals who died with VaD.^6^, ^7^ Bulk sequencing of pooled RNA isolates in VaD will not be able to resolve cells that are participating in disease progression or in tissue regeneration. However, no previous studies utilized single‐nucleus transcriptome analysis to investigate the transcriptomic changes in VaD white matter, or have revealed molecular alterations at the single‐cell level. snRNA‐seq mainly measures nuclear transcripts to profile gene expression in cells that are difficult to isolate, as well as preserved tissues. Without snRNA‐seq, the response of any cell type subpopulation (e.g., disease‐associated endothelial cell subpopulation) would be masked.

We performed single‐nucleus transcriptome analysis of VaD brain by profiling nuclei from the periventricular white matter samples of VaD patients and healthy normal control (NC) subjects. This unbiased transcriptome analysis shows that the VaD‐related, cell type‐specific transcriptomic changes in endothelial cells (ECs), microglia, astrocytes and oligodendrocytes are associated with specific molecular pathways in angiogenesis, immune response, and myelination. ECs are a particular focus of VaD^8^ therefore our approach also employed selective EC enrichment to identify EC potential heterogeneity and differential responses of these cells in VaD. Subcluster analysis of the snRNAseq data identified three types of ECs. These data identify both degenerative and regenerative events in VaD, and suggest possible therapeutic targets for disease progression and repair in this disease.

## 2 MATERIALS AND METHODS

### 2.1 Processing of brain tissue samples

Frozen post‐mortem tissue samples from the periventricular frontal white matter of vascular dementia patients and control donors without neurological disorders (NIH NeuroBioBank, UCLA Neuropathology and UC Davis). About 15 tissue samples from 5 control subjects and 5 vascular dementia (+5 vascular dementia adjacent to lesion) patients were used. The samples were age‐ and sex‐matched. (Table S1). Nearly 10 μm sections were stained with Luxol Fast Blue (Abcam, #ab150675) and affected (demyelinated) areas were excised for the vascular dementia samples. Total RNA from ~10 mg of collected tissue was isolated and used to perform RNA integrity analysis (Agilent 2100 Bioanalyzer). The samples used for nuclei isolation and single‐nucleus RNA sequencing (snRNA‐seq) had an RNA integrity number (RIN) of 6.5 ± 0.3 for NC and 5.9 ± 0.6 for VaD. An additional cohort of human brain sections was processed for immunohistochemistry, to confirm the integrity of vessels in this lesion (below).

### 2.2 qPCR

RNA was isolated from control and vascular dementia periventricular white matter (RNeasy Mini Kit (Qiagen, #74104)), cDNA synthesis performed according to published protocols,^9^, ^10^ with the following primers (all IDT [Integrated DNA Technology]): ERG (#216290168), FLT1 (#298266130), CLDN5 (#298266127), ENG (#298266133) and ACTB (#216290177). The 2^−ΔΔCt^ quantification method, using ACTB for normalization, was used to estimate the amount of target mRNA in samples, and expression calculated relative to average mRNA expression levels from control samples.

### 2.3 Nuclei isolation and snRNA‐seq using the 10X Genomics platform

Matched control and vascular dementia samples were processed in the same nuclear isolation batch to minimize potential batch effects. About 200 mg of sectioned brain tissue was homogenized in RNAase‐free homogenization buffer (250 mM sucrose, 25 mM KCl, 5 mM MgCl_2_, 10 mM Tris pH8, 1 μM DTT, 0.5× protease inhibitor, 0.2 U/μl RNaseIN, 0.1% TritonX100 and RNase‐free H_2_O) on ice. The homogenate was filtered through a 40 μM cell strainer and centrifuged at 1000 *g* for 10 min at 4°C. About 450 μl of homogenization buffer containing the nuclei was mixed with 450 μl of 50% iodixanol (Optiprep, Stem Cell Technologies, #07820). About 900 μl of 25% iodixanol containing the nuclei was layered over 900 μl 29% iodixanol and centrifuged at 13,500 *g* for 20 min at 4°C. The supernatant was discarded, and nuclei resuspended in immunostaining buffer (PBS with 0.5% BSA, 5 mM MgCl_2_, 2 U/ml DNAse and 0.2 U/μl RNaseIN). Nuclei were diluted to 1,000 nuclei/ul before performing single‐nucleus capture (10X Genomics Single‐Cell 3′ system). Target capture of 10,000 nuclei per sample was used; the 10X capture and library preparation protocol was used without modification. Single‐nucleus libraries from individual samples were pooled and sequenced (NovaSeq S2, average depth 70,000 reads/nucleus).

### 2.4 Nuclei FACS sorting

After nuclei isolation and prior to nuclei capture, the nuclei were FACS sorted using an anti‐ERG antibody conjugated to Alexa Fluor 555. This antibody cross reacts with FLI1^11^ also named ERGB. FLI1 is expressed in microglia and endothelial cells, both from a common embryonic origin,^9^, ^10^ to enrich samples in endothelial and microglial nuclei.^11^ The nuclei were incubated with the antibody in immunostaining buffer on a rotator in cold room for 40 min. The nuclei were washed once with 500 μl washing buffer (PBS with 0.5% BSA, 5 mM MgCl_2_, 2 U/ml DNAse) and centrifuged at 400 *g* for 5 min at 4°C. The nuclei were collected during FACS sorting in immunostaining buffer.

### 2.5 snRNA‐seq data processing with 10X genomics CellRanger software and data filtering

A pre‐mRNA reference genome according to the instructions provided by 10× Genomics was generated. The demultiplexed FASTQ files (Novogene) to the GRCh38 pre‐mRNA reference genome were subsequently aligned (Cell Ranger, 3.0.1).^6^, ^12^ Individual expression matrices containing unique molecular identifiers per nucleus per gene were used as input in downstream analysis (Seurat, 3.0).^13^ Cells with a number of expressed genes <500 or where the percent of mitochondrial genes was over 5% of total expressed genes were removed, as was the potential doublets that occurred in the encapsulation step (DoubletFinder package, 2.0.2).^14^ A total of 172,943 filtered nuclei were used for further bioinformatic analysis.

### 2.6 Data integration and dimensionality reduction

The gene expression data from individual samples were processed (Suerat, Read10× () function). For each sample, gene expression was represented as the fraction of the gene and multiplied by 10,000, converted into natural logarithm and normalized after adding 1 to avoid taking the log of 0. The top 3000 highly variable genes (HVGs) from the normalized expression matrix were identified, centred and scaled before we performed principal component analysis, based on these HVGs. The batch effects were removed (Harmony package 1.0) on the top 50 PCA components identified.^15^

### 2.7 Cell‐clustering and annotation

Clustering analysis was performed on the integrated joint embedding (Harmony) with the Louvain algorithm after computing shared nearest‐neighbour graph with the Louvain algorithm that (“FindClusters”, Seurat). Initial cluster annotation was done (scCATCH package, 2.0).^16^ In parallel, DEGs with high discrimination abilities between the clusters were identified (“FindAllMarkers”, Seurat) using the default non‐parametric Wilcoxon rank sum test with Bonferroni correction. The cell types were annotated based on the DEGs and the well‐known cellular markers from the literature.

### 2.8 Examination of cell type‐specific transcriptomic changes

Vascular dementia samples were stratified according to the initial classification of VaD, VaDadj and NC samples and compared with the transcriptome profiles of individual cell types between VaD and NC and VaDadj and NC by Wilcoxon rank‐sum test (FindMarkers function, parameters logfc.threshold = 0 and test.use = wilcox).

### 2.9 Subcluster analysis

Individual cell types were isolated from the original Seurat dataset using the Subset function. Subsequently, each cell type was reclustered using an approach similar to that used for the initial cell type clustering. ‘K’‐nearest neighbour clustering using the ‘FindClusters’ function with the parameters ‘resolution = 0.1’ and UMAP clustering using the ‘RunUMAP’ function with the parameter ‘dims = 1:20’ was performed. Transcriptomic signatures were identified with the ‘FindMarkers’ function. The level of statistical significance was set at an adjusted *p* < 0.001, log fold change ≥0.25 or ≤−0.25. For WAM/DAM signature gene enrichment analysis, FindAllMarkers function was used with 10% minimum percent of cells expressed on microglia subclusters. Rank‐Rank Hypergeometric Overlap (RRHO) tests against several white matter and neurodegenerative disease microglial data sets^17^, ^18^ by ranking genes by logFC values for each microglia cluster.

### 2.10 Human brain immunohistochemistry

Formalin fixed post‐mortem tissue samples from the periventricular frontal white matter of vascular dementia patients were obtained from NIH NeuroBioBank. As with the snRNAseq samples, these samples were diagnosed with no Alzheimer's disease or other type of dementia in pathological studies.

10% formalin fixed tissue samples were processed (UCLA Translational Pathology Core Laboratory), embedded in paraffin and microtome‐sectioned into 7‐µm thick slices. Briefly, the slices were rehydrated in 3× wash of xylene followed by gradient wash of alcohols (2× 95%, 80% and 70%) and TBS. To expose the epitopes caused by paraffin fixation, the slices were bathed in sodium citrate buffer (pH 6.0) in 95°C for 20 min. The endogenous peroxidase was blocked by incubation of the slices in 3% hydrogen peroxide for 10 min in room temperature. Primary antibodies: glucose transporter 1 (GLUT1) (MilliporeSigma, cat# 07‐1401, 1:1000, rabbit antibody) as a specific endothelial cell marker, endoglin (ENG) (R&D System, cat# AF1097, 5 µg/ml, goat antibody), vascular endothelial growth factor receptor 1 (FLT1) (R&D System, cat# AF321, 20 µg/ml, goat antibody), tight junction protein claudin‐5 (CLND5) (Abcam, cat# ab15106, 1:30, rabbit antibody), ERG (Abcam, cat# ab92513, 1:30, rabbit antibody) were used to validate the specific expression of deregulated genes in endothelial cells; HRP conjugated goat and rabbit secondary antibodies used were from Vector Laboratories, ref# MP‐7405 and MP‐7401. The target proteins were visualized by chromogen development (NovaRED and DAB, Vector Laboratories, ref# SK‐4800 and SK‐4100). Mono colour immunohistochemistry in consecutive slices was applied when the antibodies of target genes were produced from rabbit, the same as GLUT1 antibody; while dual colour staining on the same brain slice was applied when antibodies of target genes were from different species. NovaRED produces a red deposit, DAB gives a brown deposit. The colocalization of two antibodies will appear reddish‐brown. The brain sections were counterstained with haematoxylin to show nuclei. Slides were scanned and digitized (Panoramic Midi 2, Epredia).

## 3 RESULTS

### 3.1 Endothelial and microglial nuclei sorting and single‐nucleus transcriptomic profiling of the periventricular white matter in VaD

**FIGURE 1:**
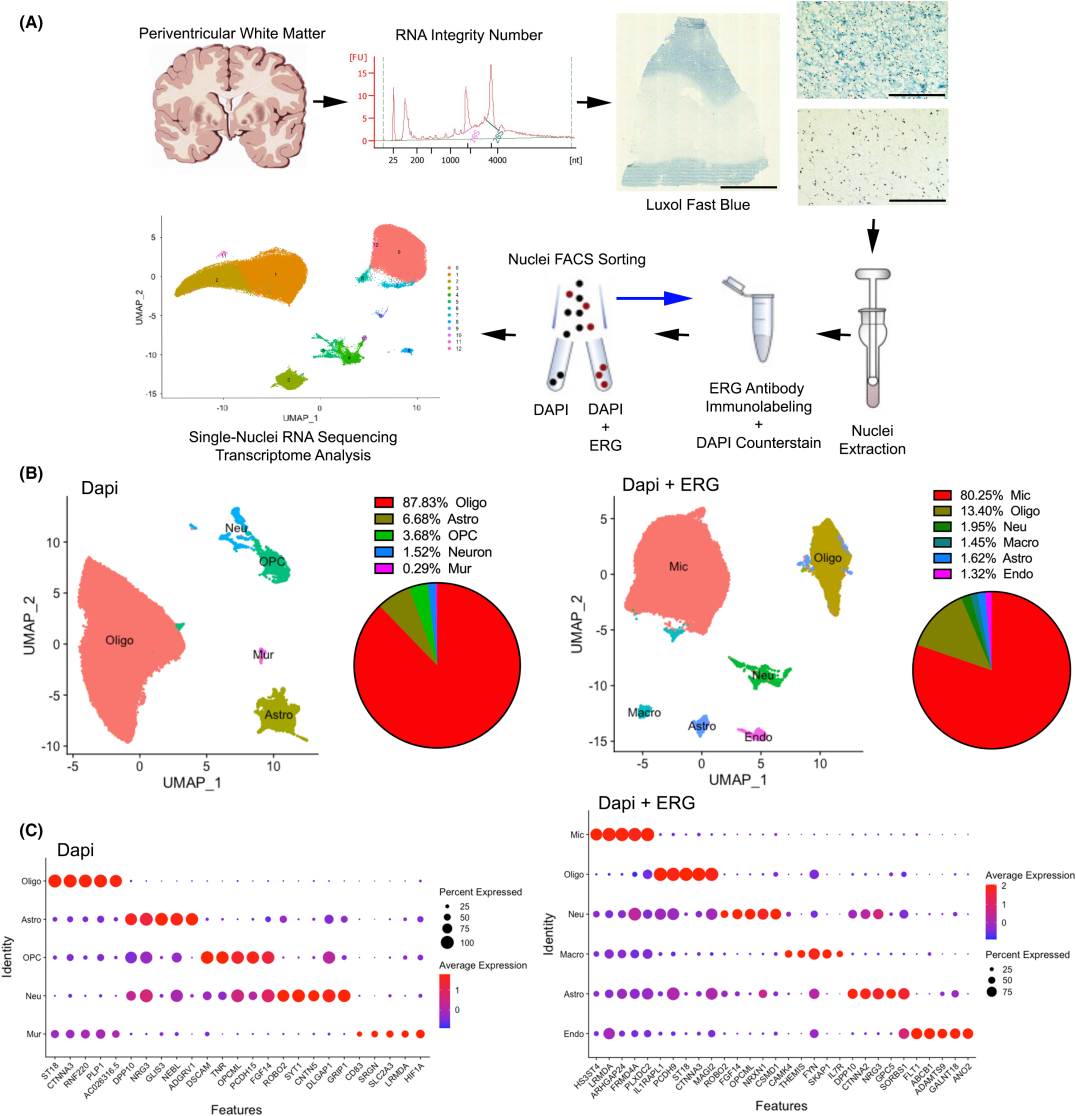
Single‐nucleus transcriptome analysis of the PVWM in VaD. (A) Single‐nucleus transcriptome profiling workflow. Scale bar 5 mm and 200 μm. (B and C) Unbiased identification of cell‐type heterogeneity in the human PVWM. (B) UMAP plot showing the 5 (DAPI condition) and 6 (DAPI + ERG condition) major cell types isolated from PVWM and proportions of cell types among the 172,943 sampled nuclei. (C) Dot plot showing the top five most enriched genes for each cell type. See also Figures S1–S3 and Tables [Link], [Link], [Link], [Link]

To investigate the molecular and cellular profiles of brain tissues alterations in patients with VaD compared to those in healthy normal control (NC) subjects, we performed transcriptome analysis of 15 periventricular white matter (PVWM) tissue samples from patients with VaD (lesion *n* = 5 and adjacent to the lesion *n* = 5), and NC subjects (*n* = 5) (Table S1) at the single‐cell level by single‐nucleus RNA sequencing (snRNA‐seq). There were no significant differences in age, gender balance or postmortem interval across samples. The VaD samples did not evidence AD pathology, such as amyloid beta accumulation or tau pathology. The PVWM near the frontal horn is the site of the most common white matter lesions in VaD.^19^ Sections from human tissue blocks contain the frontal PVWM, and this gene expression analyses were targeted at this region. (Figure 1A). The quality of the sample is one of the key steps in obtaining reliable data; therefore, we assessed the RNA integrity number (RIN) from 42 PVWM tissue samples and chose 10 with the highest RIN (Table S1). The samples used for nuclei isolation and single‐nucleus RNA sequencing (snRNA‐seq) have an RNA integrity number (RIN) of 6.5 ± 0.3 for NC and 5.9 ± 0.6 for VaD. We next stained 10 um slices for myelin with Luxol fast blue and identified the affected (demyelinated) areas in VaD tissue samples (Figure 1A), defined as a pallor in white matter myelin staining.^20^, ^21^ We then separated the demyelinated areas (referred as VaD) from adjacent white matter (referred as VaDadj) (Figure S1) and used both for nuclei isolation together with the NC tissue samples. To specifically enrich our samples in endothelial cells and microglia, with their important role in dementia and white matter disease,^22^ we immunolabeled the nuclei using an antibody against ERG/FLI1 (EPR3864) and FACS sorted these cells. After the FACS analysis, we classified the 2 two conditions as DAPI (only DAPI positive nuclei) and DAPI +ERG (DAPI and ERG positive nuclei). The two isolates were analysed separately. In total, we sampled 172,943 nuclei: 53,156, 59,447 and 60,340 nuclei from VaD, VaD adjacent to the PVWM lesion (VaDadj) and NC brain samples respectively. The sample quality was checked again by the mean numbers of transcripts and genes detected per nucleus (Figure S2A).

To establish a baseline profile of cell populations, we performed initial unbiased projection (UMAP) clustering on all 30 samples (DAPI and DAPI + ERG conditions) from VaD, VaDadj and NC subjects. This analysis yielded 8 clusters for DAPI condition and 7 unique cell clusters for the DAPI + ERG condition, which we subsequently categorized based on sets of cell‐type related markers into the following 5 and 6 major cell types (scCATCH)^16^ (Figure 1B and Table S2), a cluster‐based automatic annotation approach, where cell types are scored on the basis of validated marker genes, and the cell type with the highest score (top 1) is determined for the cluster. In addition, cell clusters were manually verified and annotated according to their individual transcriptome profiles and previously reported cell‐type: oligodendrocytes (MBP^+^, 86.4 ± 3.4% of total nuclei), astrocytes (AQP4^+^, 7.0 ± 1.4%), oligodendrocyte progenitor cells (OPC) (VCAN^+^, 4.3 ± 0.6%), neurons (RBFOX3^+^, 1.7 ± 1.6%), mural cells (PDGFRB^+^, 0.3 ± 0.0%) for the DAPI condition and microglia (CSF1R^+^, 76.6 ± 3.9%), oligodendrocytes (PLP1^+^, 16.4 ± 3.3%), neurons (RBFOX3^+^, 2.0 ± 0.8%), macrophages (PTPRC^+^, 1.5 ± 0.2%), astrocytes (AQP4^+^, 1.6 ± 0.5%), endothelial cells (CLDN5^+^, 1.5 ± 0.3%) for DAPI +ERG condition (Figure 1B and Figure S2B,C). The successful selection for endothelial cells and microglia enrichment in the ERG/FLI1 population is confirmed by the near absence of cells with these markers in the DAPI condition and the expression of ERG and FLI1 only in the DAPI + ERG condition (Figure S3). In addition to the previously reported cell‐type markers, major cell types expressed the following unique signature genes: ST18 and CTNAA3 were expressed by oligodendrocytes; GLIS3 and ADGRV1 by astrocytes; HS3ST4 and LRMDA by microglia; and ABCB1, ADAMTS9 and GALNT18 by endothelial cells (Figure 1C and Tables S3 and S4). These results collectively and comprehensively reveal that this approach captures the major cell types of the subcortical white mater in diseased and healthy PVWM tissues.

### 3.2 Cell type‐specific transcriptomic changes reveal dysregulated molecular pathways in VaD PVWM

**FIGURE 2:**
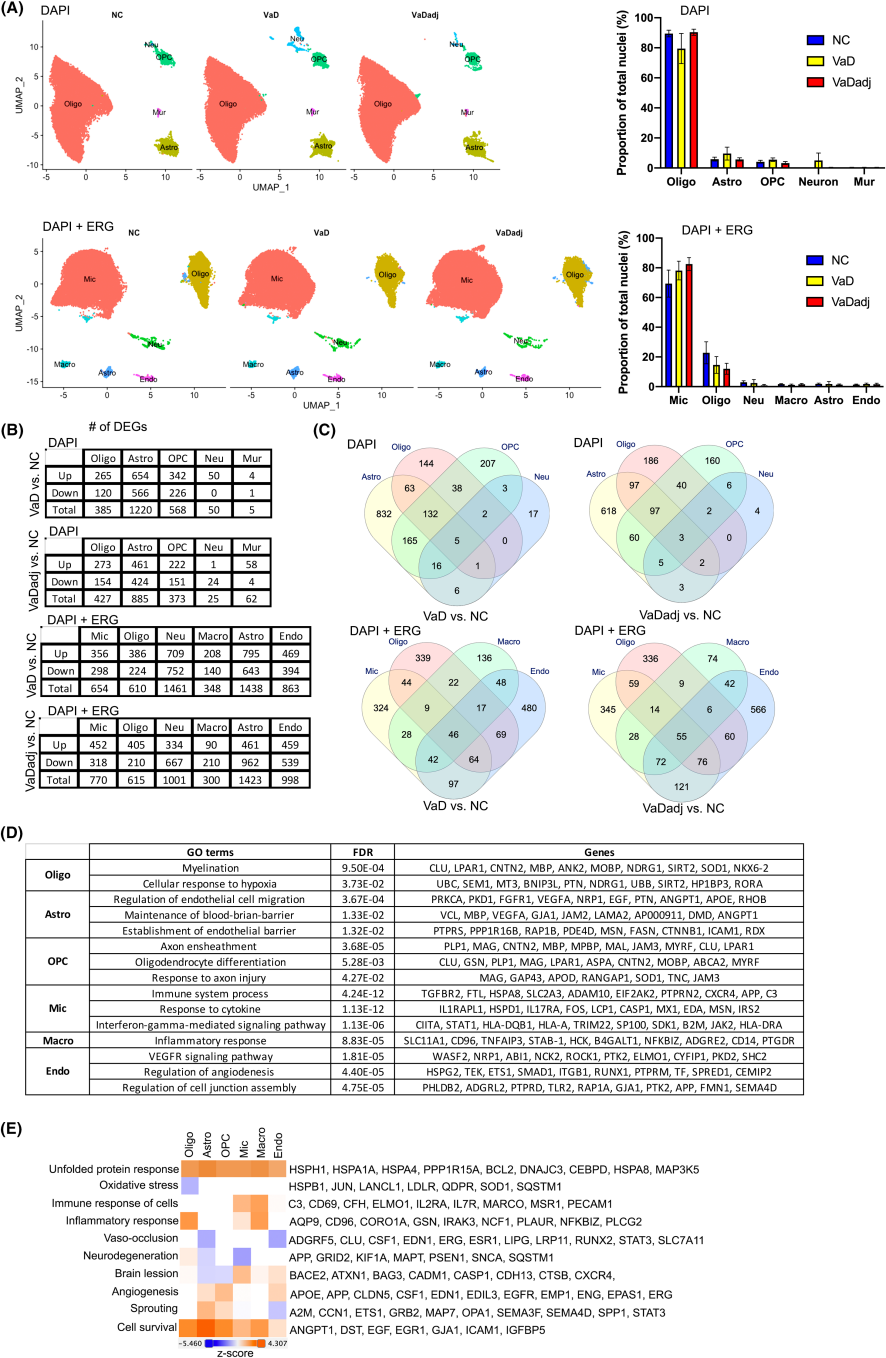
Dysregulated molecular pathways in VaD according to cell type‐specific transcriptomic changes. UMAP plots and bar plots (A) showing the proportions of the major cell types found in the VaD, VaDadj and NC PVWM samples. (B and C) VaD and VaDadj‐ associated transcriptomic changes were highly cell type‐specific. (B) Numbers of DEGs between VaD, VaDadj and NC samples within each cell type (adjusted *p* < 0.001, log fold change ≥0.25 or ≤−0.25). Down: down‐regulated; Up: up‐regulated. (C) Venn diagram showing the coregulated DEGs among cell types. (D and E) the cell type‐specific transcriptomic changes in VaD were associated with distinct molecular pathways. (D) Table showing the specific GO terms in cell types in VaD samples. (E) Heatmap showing molecular pathways activation using Ingenuity Pathway Analysis (IPA) software. Astro, astrocytes; Neu, neurons; OPC, oligodendrocyte progenitor cell; Oligo, oligodendrocyte; Mur, mural cells; Endo, endothelial cells; Mic, microglia; Macro, macrophage. All data are mean ± SEM. See also Figures S4 and S5 and Table S5

Following initial cell‐type characterization, we compared the proportions of different cell types between VaD, VaDadj and NC PVWM samples. UMAP cluster analysis revealed that the proportions of oligodendrocytes tend to decrease from 89.5% in NC to 79.5% in VaD, astrocytes slightly increased from 5.9% in NC to 9.6% in VaD, microglia increased from 69.4% in NC to 78.1% in VaD to 82.5% in VaDadj, and OPC, neurons, mural cells, macrophages and endothelial cells are similar in the isolated samples among VaD, VaDadj and NC PVWM samples (Figure 2A).

To examine the global transcriptomic changes in individual PVWM cell types in VaD and VaDadj, we compared the individual cell‐type transcriptome profiles between VaD vs. NC and VaDadj vs. NC samples using a level of statistical significance at an adjusted *p* < 0.001 and log(2) fold change ≥0.25 or ≤−0.25. We identified 2,228 (VaD vs NC PVWM samples) and 1772 (VaDadj vs NC PVWM samples) differentially expressed genes (DEGs) in the DAPI condition, and 5374 (VaD vs NC PVWM samples) and 5,107 (VaDadj vs NC PVWM samples) in the DAPI + ERG condition (Figure 2B and Table S5). Among the DEGs, very few are differentially expressed in all cell types (Figure 2C), suggesting that most observed VaD‐associated transcriptomic changes are cell type‐specific. The greatest difference in gene expression is between VaD and NC, and a more detailed analysis of the transcriptomic differences between these two conditions follows.

An analysis of significantly regulated genes in VaD by cell type and gene ontology (GO) indicates that there are specific molecular processes significantly regulated by cell type in VaD. (Figure 2D and Figures S4 and S5). The DEGs (VaD vs NC) in endothelial cells (EC) (e.g., APP, ROCK1, PTK2, ELMO1, ETS1 and RUNX1) are associated with VEGF signalling and regulation of angiogenesis and cell junction assembly. Similarly, the DEGs (VaD vs NC) in astrocytes (e.g., VEGFA, ANGPT1 and ICAM1) are associated with regulation of endothelial cell migration and maintenance of blood‐brain barrier. Besides the transcriptomic changes in endothelial cells and astrocytes, GO analysis also reveals that the DEGs in oligodendrocytes (e.g., LPAR1, MBP and MOBP, Figure S6), OPC (e.g., MAG, APOD and TNC), microglia (e.g., FTL, CXCR4 and C3) and macrophages (e.g., SLC11A1, CD96 and NFKBIZ) were associated with myelination, response to axon injury, immune response and inflammatory response respectively (Figure 2D). The neurons and mural cells sets are very small in this white matter isolation and have no gene GO term in this approach. These results indicate that in VaD, each cell type in the white matter has specific pathways that are significantly regulated in their gene expression profile. These pathways relate to mechanisms of injury response or repair in each cell's function, such as myelination and axonal signalling in cells of the oligodendrocyte lineage, specific inflammatory pathways in microglia and macrophages and a dual angiogenic signalling ontology in both astrocytes and endothelial cells.

Gene ontology analyses produce results in broad biological categories. To better define the specific signalling systems in VaD by cell type, the differentially expressed genes were analysed according to molecular pathway. Molecular pathway analysis indicates a coincident induction of genes associated with angiogenesis. There is an increase in angiogenesis in endothelial cells, astroglia and OPC (Figure 2E and Table S5) (e.g., APP, CLDN5, CSF1 and EGFR). This induction of angiogenesis genes is not uniform, as endothelial cells show gene expression patterns also that link to an inhibition of sprouting in VaD (e.g., A2M, MAP7, OPA1 and SEMA3F). Interestingly, all the cell types show an activation of cell survival process with endothelial cells having the weakest activation of cell survival gene sets when compared to the other cell types. Finally, there is an activation of unfolded protein response in all the cell types in VaD (eg., HSPH1, HSPA4 and DNAJC3) (Figure 2E and Table S5).

Together, our results reveal that the cell type‐specific transcriptomic changes in VaD are associated with the following molecular pathways: angiogenesis and sprouting cells in endothelial cells and astroglia, immune and inflammatory response in microglia and macrophages, myelination in oligodendrocytes, and response to axon injury in OPC.

### 3.3 Microglia subclustering identifies aging and disease gene signatures

**FIGURE 3:**
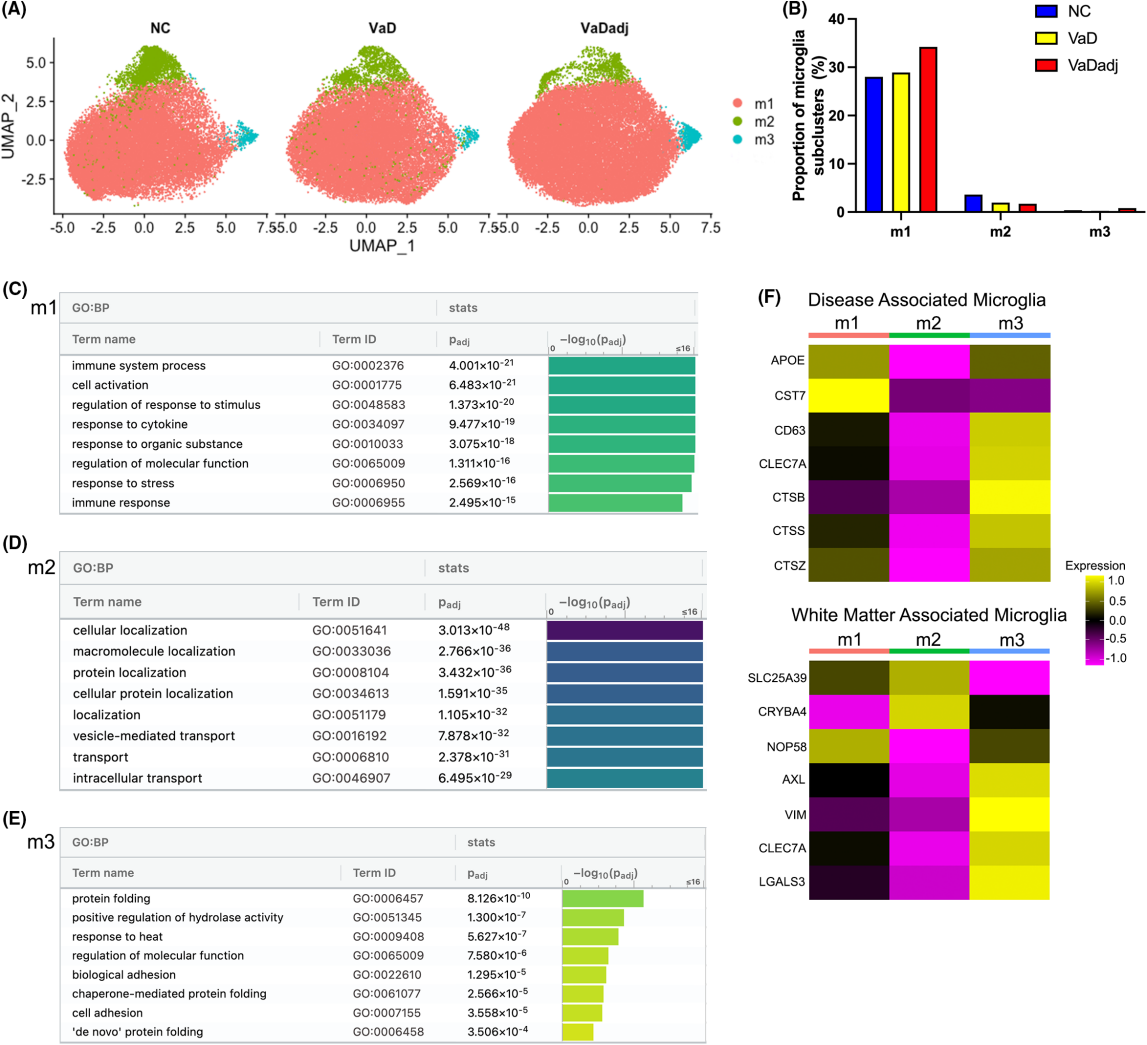
VaD microglia subclusters show enriched genes of immune and cytokine responses and disease associated gene signature. (A) UMAP plots showing the distribution of microglia subpopulations (m1–m3) in VaD, VaDadj and NC PVWM samples. (B) Proportion of microglia subclusters. (C–E) GO pathway analysis (g:Profiler) of the transcriptome signature of microglia subclusters (m1–m3). (F) Disease‐associated and white matter‐associated microglia gene signatures. See also Tables S6 and S7

Vascular dementia lesions are tightly associated with local microglial activation^17^ and interaction with endothelial cells.^18^ For these reasons, we deliberately selected for endothelial cells and microglia in a parallel snRNA‐seq process. We performed subcluster analysis on this enriched FACS isolate of endothelial cells and microglia and determined their subpopulation heterogeneity. Unbiased projection (UMAP) subclustering analysis of microglia, in all three conditions, identified 3 transcriptionally unique subpopulations (Figure 3A). The relative proportions of subpopulations m1, m2 and m3 were similar between the VaD, VaDadj and NC samples (Figure 3B). m1 is the main microglial subcluster followed by 2 small subclusters which have, by gene expression analysis, different roles in the brain. Using a level of statistical significance at an adjusted *p* < 0.001 and log fold change ≥0.25 or ≤−0.25, the three microglial subpopulations expressed specific DEGs when VaD is compared with NC (Table S6). GO enrichment analysis by g:Profiler in Biological Processes (BP)^23^ reveals that m1 is characterized by enriched expression of genes associated with immune response and response to cytokines (i.e., IFI44, ITGAV, EIF2AK2, CSF3R, CX3CR1 and HLA‐DRA) while m2 has an enriched expression of genes in cellular and protein localization (i.e., TIAM1, MSR1, MAP7 and TNFRSF1B) (Figure 3C,D and Table S6). The m3 subcluster is defined by genes involved in protein folding, including DNAJB1, HSPH1, PTGES3, DNAJB6, FKBP4 and HSPD1, indicating a stress response (Figure 3E and Table S7).

Recently, subsets of microglia have been defined based on aging‐ and disease‐association. Microglia undergo a defined alteration in gene expression associated with damage or degeneration, defined as disease‐associated microglia (DAM).^17^, ^18^, ^24^ In the aged white matter, single cell RNAseq has identified a unique microglia group.^17^ The gene expression profile of these three subclusters of microglia were compared with the signature genes of DAM and white matter‐associated microglia (WAM).^17^, ^18^, ^24^ A heatmap was generated with the average expression of these genes in each of the microglial subclusters (Seurat) and scaled for each gene (Figure 3F). Interestingly, the m3 subcluster expresses genes that were previously identified in WAM (e.g., AXL, VIM, CLEC7A and LGALS3) and DAM (e.g., CD63, CLEC7A, CTSB, CTSS and CTSZ) (Figure 3F).^1^, ^25^ Rank‐rank hypergeometric overlap analysis (RRHO) tests show that m2 cluster marker genes are significantly overlapping with those of both activated (ACT) and white matter associated microglia (WAM) microglial phenotypes^17^ and to microglia associated with amyloid plaques in Alzheimer's disease models and DAM1/2^18^, ^24^ (Table S8). DAMs have the potential to restrict disease progression and damage by enhancing clearance of misfolded and aggregated proteins that commonly accumulate in neurodegenerative diseases^26^, ^27^ suggesting a response to the diseased environment in white matter VaD.

### 3.4 Heterogeneity of endothelial cell subpopulations contributes to disease progression and tissue repair

**FIGURE 4:**
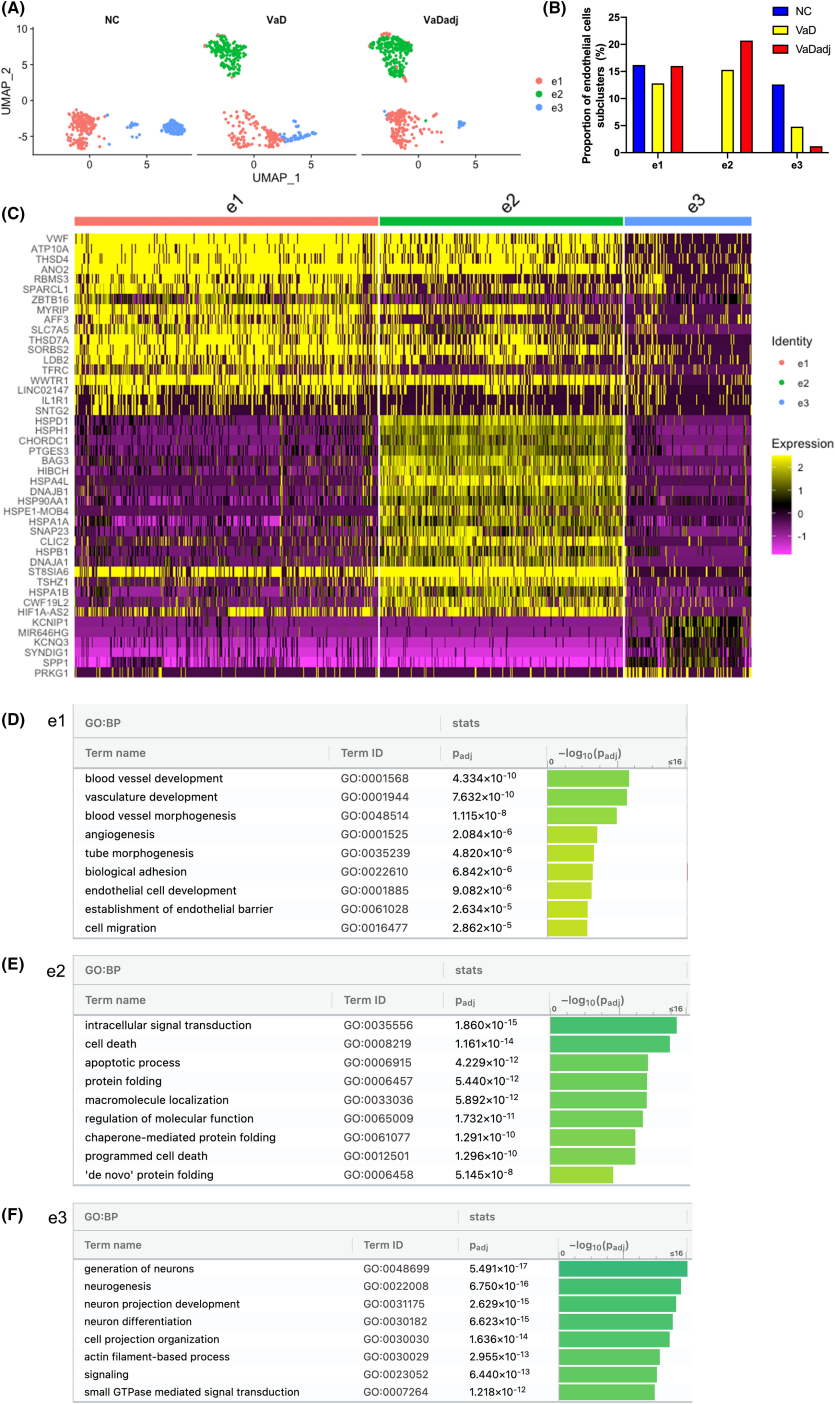
A distinct endothelial cell subpopulation is present in VaD. (A–C) Transcriptomically unique subpopulations of endothelial cells were present in VaD samples. (A) UMAP plots showing the distribution of endothelial subpopulations (e1–e3) in VaD, VaDadj and NC PVWM samples. (B) Distribution of VaD‐associated endothelial subpopulations. (C) Expression levels of the top enriched genes in the three subpopulations at the single‐cell level (adjusted *p* < 0.001, log_2_ fold change >0.25). (D–F) GO pathway analysis (g:Profiler) of the transcriptome signature of the three endothelial cell subclusters (e1–e3). See also Tables S9 and S10

**FIGURE 5:**
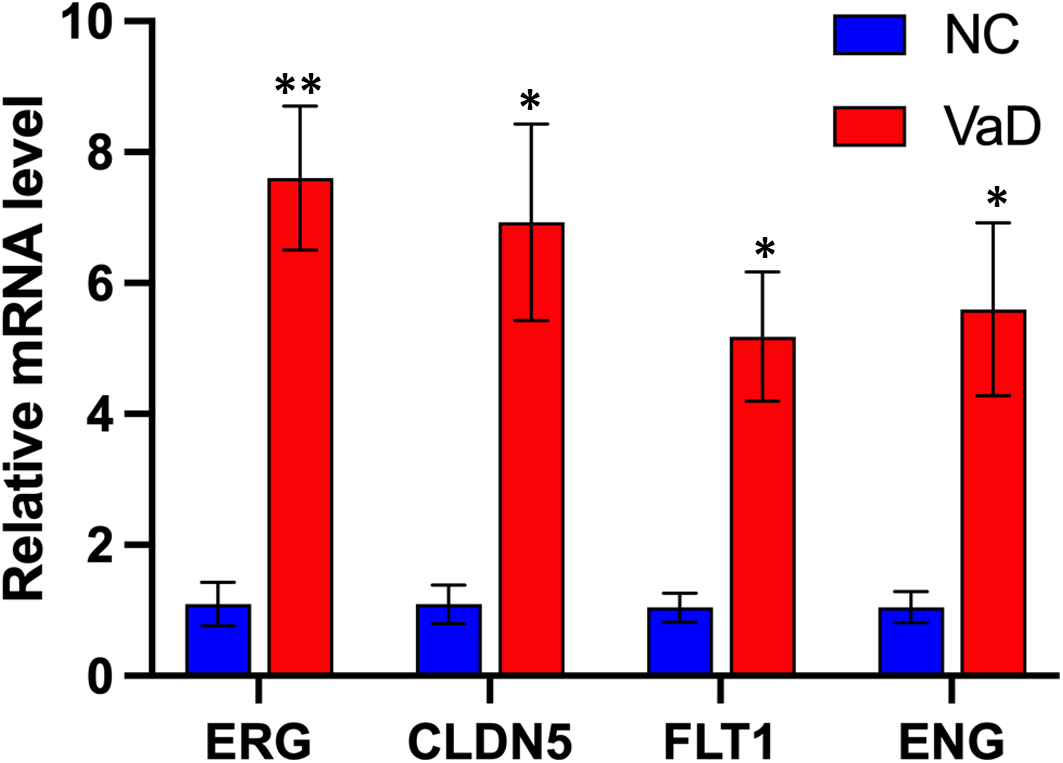
Validation of endothelial key DEGs. Indicated genes were analysed by quantitative PCR (qPCR). Expression is shown relative to mean expression of NC (*n* ≥ 3, mean ± SEM, unpaired Student's *t*‐test, **p_ERG_ = 0.0045, *p_CLDN5_ = 0.0224, *p_FLT1_ = 0.0174, *p_ENG_ = 0.0432)

A central cellular focus for vascular dementia is the endothelial cell.^28^, ^29^ Through all three conditions, unbiased clustering of endothelial cells by differentially regulated genes identified 3 different subpopulations (e1, e2 and e3). The e2 subcluster is present only in cases of dementia: VaD and tissue adjacent to VaD lesions (Figure 4A,B). Comparison of the most differentially expressed genes in these three subclusters clearly shows their segregation into distinct gene sets by expression change localized to each cluster (Figure 4C and Table S9). The endothelial cells in the VaD‐unique subcluster e2 (disease associated) has genes within ontology classes that relate to cell stress and disease, such as apoptosis, protein folding, chaperone function and cell death^30^ (i.e., HSPD1, HSPH1 and BAG3) (Figure 4E and Table S10). Clusters e1 and e3 are present in both NC and VaD but their differential gene expression in VaD compared with NC reflect endothelial responses to VaD (Figure 4D,F and Table S10). These are organized into two broad ontological classes of blood vessel and vascular development and angiogenesis (i.e., VWF, ANO2, CLDN5, ERG and FLT1) (e1); and neurogenesis, neuron projection development and neuron differentiation (i.e., SLC1A3, SPP1, SEMA4D, APOE, SYT1 and SYT17) (e3). Most of the secreted or receptor/ligand gene products in the neuronal molecular pathways signal to OPCs, and are linked to OPC differentiation^31^ (TIMP2, SEMA4B, neuroligin/neurexins, NCAM1, NCAM2, ROBO1 and UNC5C) (Table S10). This is an interesting distinction of two clusters of ECs in VaD with two different regeneration responses: angiogenesis and axonal/OPC regeneration. Of these significantly regulated genes in the e3 cluster by snRNA‐seq, we confirmed that several key DEGs were indeed upregulated by qPCR in VaD; CLDN5, FLT1, ENG and ERG by qPCR (Figure 5). Of note, these confirmed genes are also used as endothelial cell markers,^24^, ^29^, ^30^, ^32^ as they are specifically expressed in endothelial cells (Figure S7). The activation of these gene classes in endothelial cells in VaD indicate distinct populations of angiogenic and neuronal remodelling/OPC differentiation with beneficial and degenerative responses in VaD.

## 4 DISCUSSION

The identification of precise molecular and cellular targets for VaD therapeutic development requires a comprehensive understanding of the cell type‐specific responses and cellular heterogeneity in VaD. Single nucleus RNAseq from fresh‐frozen human brain samples of VaD allows specific targeting of each cell types of the most affected part of the brain in VaD, the periventricular white matter. Further, our approach specifically focused on endothelial and microglial cells using an additional FACS isolation step, as endothelial cells are often not well isolated in standard single cell RNA studies.^33^ We used myelin staining of the tissue block to direct the sampling of cells from the lesion site in VaD and the adjacent white matter to the lesion, versus subjects with normal white matter.

Single‐nucleus transcriptome analysis identified eight major molecular pathways that are dysregulated in in VaD. These are in an increased unfolded protein response in all cell types, reduced myelination in oligodendrocytes, increased inflammatory responses in microglia/macrophages, increased axon ensheathment in OPCs, enhanced angiogenesis and sprouting inhibition in endothelial cells and regulation of cell migration in astroglia. Moreover, we showed that the dysregulation of the pathways in VaD endothelial cells is due to changes in subpopulations of these cells, including the appearance of a new subpopulation in VaD which is associated with increased cell death and apoptosis. Two molecular responses emerge in two different classes of endothelial cells, with differentially expressed genes having common biological functions in angiogenesis and axonal/OPC regeneration. Together, this single‐nucleus transcriptomic profiling highlights the potential roles of both progressive degeneration and a regenerative response in the glial and endothelial cells of the white matter in VaD.

The results of our single‐nucleus transcriptomic profiling of VaD white matter are a useful resource for understanding the cellular dysregulation along different cell types. For example, we show that there is an increase in the unfolded protein response in all cell types in the VaD white matter and a dysregulated pathway in oligodendrocyte myelination process. These findings are similar to other neurodegenerative diseases, such as ALS, Parkinson's and Alzheimer's disease, in which the unfolded protein response is both part of the reaction to the progressive damage, and may also be part of the active spread of the disease.^31^, ^33^, ^34^, ^35^

This transcriptome profiling reveals an aberrant immune activation and a response to cytokines in microglia. Neuroinflammation plays a prominent role in the pathogenesis of vascular dementia^26^, ^36^ and the present results show the dysregulation of specific gene systems with a role microglial function and neurodegenerative disease, such as FTL, CXCR4 and C3.^37^, ^38^, ^39^ Subsets of microglia in VaD share gene expression patterns previously reported for white matter microglia^17^ and for microglial gene activation around amyloid plaques—damage associated microglia.^18^, ^24^ The data in the present study confirm that, as in other neurodegenerative diseases, there is a subset of microglia in VaD that react to the damage and may limit further spread.

The endothelial cell in VaD is a central element of the disease process. The main risk factor for VaD, hypertension, affects the microvasculature of the brain and particularly periventricular white matter, to drive this disease. However, primary endothelial cell dysfunction also plays a role in VaD, with evidence from animal modelling and human GWAS studies indicating primary genetic risk in endothelial cells.^29^ The present results identify a unique endothelial subcluster present only in VaD brains associated with enhanced gene expression in protein folding and programmed cell death responses. Activation of the unfolded protein response is as a common pathway in protein‐misfolding neurodegenerative diseases, with relevant markers observed in patient tissue and mouse models.^3^, ^26^, ^35^ The concept of disrupted protein homeostasis through endoplasmic reticulum (ER) stress and activation of the unfolded protein response (UPR) is a major common pathogenic process in many neurodegenerative diseases. Because the UPR may be pathologically triggered and involved not just in a cell's reaction to damage‐inducing signals, but a maladaptive response that promotes disease progression and the UPR has emerged as a target for therapy,^35^, ^37^ which the present data shows may apply to VaD.

In addition to the signals within endothelial cells of VaD for disease progression, there is a robust induction of two different regenerative responses. Two distinct clusters of endothelial cells, identified by unique patterns of gene expression, respond with angiogenesis and axonal and OPC regenerative responses. In rodent models of VaD, global cerebral hypoperfusion induces angiogenic factors and cell surface molecules, such as connexin‐43, which produce angiogenesis.^38^, ^39^ In human patient populations with dementia or mild cognitive impairment, the pro‐angiogenic growth factors are associated with both early stages of the disease process^40^ and disease progression.^41^, ^42^ CLDN5, FLT1, ENG and ERG localization to and upregulation within the vasculature in VaD was confirmed by qPCR and immunohistochemistry. ERGhas emerged as a major regulator of endothelial function, promoting angiogenesis.^43^ CLDN5 regulates BBB permeability.^44^ ENG has many roles in cerebrovascular diseases,^45^ and FLT1expression inhibits abnormal brain angiogenesis^46^ endorsing the importance of these gene expression changes in vessel reaction to and possibility contribution to disease progression or repairs. Moreover, another single‐nucleus transcriptome analysis study found a dysregulation of angiogenic endothelial cells showing an increased expression of FLT1 in AD brains.^47^ In a disease with loss of vascular function and density, the substantial and coordinated upregulation of an angiogenic response in a subset of endothelial cells indicates a possible regenerative action in the white matter microvasculature. In light of these conclusions, it is worth noting some of the limitations of snRNAseq or scRNAseq. The sequencing depth of each cell is not as deep as batch RNAseq approaches. This is usually made up for in the aggregate sequencing of thousands of cells; however, it can limit depth of gene expression analysis in a given cell type. The use of snRNAseq in human archival samples is well established, but is limited potentially by RNA quality issues in frozen and stored human samples. These quality issues were directly assessed in the present study.

Inflammatory white matter diseases, such as multiple sclerosis, are associated with robust regeneration in OPCs, re‐myelination and enhanced axo‐glial signalling in their early phases.^48^ However, ischaemic white matter disease is associated with local progression into initially normal‐appearing white matter.^49^, ^50^ Endothelial signalling to OPCs and white matter myelination is a strong component of normal brain development.^51^ In mouse models of vascular dementia, specific gene systems block OPC differentiation and inhibit white matter repair, which can be targeted and reversed.^52^, ^53^ The present transcriptional profiling of endothelial cells in human VaD identifies genes systems that are in a position to signal between endothelial cells, OPCs or glial progenitors and adjacent axons and promote OPC survival to stress, differentiation and myelination. These include SPP1, anosmin‐1, neurexins 1 and 3, neuregulin 2, SEMA4D, TIMP2 and ROBO1.^4^, ^54^, ^55^, ^56^, ^57^, ^58^, ^59^, ^60^, ^61^ It is possible that a limited or restricted program of white matter repair, characterized by endothelial‐OPC‐axonal signalling, is present in human VaD and may serve as a target for therapies to promote regenerative responses.

In summary, the present transcriptome profiling results constitute a resource for understanding the pathological roles of the white matter cells in VaD and a central role of the endothelial cell is both disease progression and tissue regeneration. The white matter neurovascular niche is a complex interplay of intercellular signalling systems that are in a position to control the disease and its repair.

## CONFLICT OF INTEREST

The authors confirm that there are no conflicts of interest.

## AUTHOR CONTRIBUTIONS

**Daniel Nicolae Nicolae Mitroi:** Conceptualization (equal); Data curation (equal); Formal analysis (equal); Investigation (equal); Methodology (equal). **Riki Kawaguchi:** Data curation (equal); Formal analysis (equal); Methodology (equal). **William E Lowry:** Conceptualization (equal); Data curation (equal); Formal analysis (equal); Funding acquisition (equal); Methodology (equal). **Stanley Thomas Carmichael:** Conceptualization (equal); Data curation (equal); Formal analysis (equal); Funding acquisition (equal); Methodology (equal); Project administration (equal). **Min Tian:** Formal analysis (equal); Methodology (equal); Writing – original draft (equal).

## Supporting information

Fig S1‐S7

Tab S1

Tab S2

Tab S3

Tab S4

Tab S5

Tab S6

Tab S7

Tab S8

Tab S9

Tab S10
