# Supplementary material for: Single‐nucleus transcriptome analysis reveals disease‐ and regeneration‐associated endothelial cells in white matter vascular dementia
Source: J Cell Mol Med. 2022 May 11;26(11):3183–95. doi: 10.1111/jcmm.17315 (PMC9170821; doi:10.1111/jcmm.17315)
Supplement: Supplementary file 1 — Fig S1‐S7 [file JCMM-26-3183-s002.pdf]

Supplementary Figures for Mitroi et al.

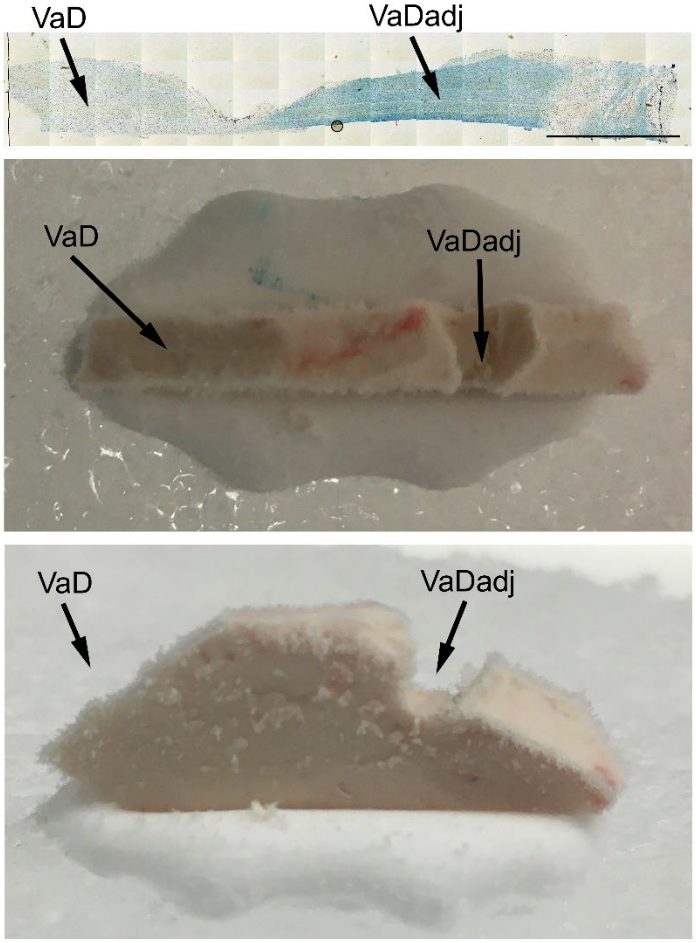

Sup Fig 1. Identification of demyelinated area (referred as VaD) from adjacent white matter (referred as VaDadj) using Luxol Fast Blue stain kit (related to Figure 1). Scale bar 5 mm.

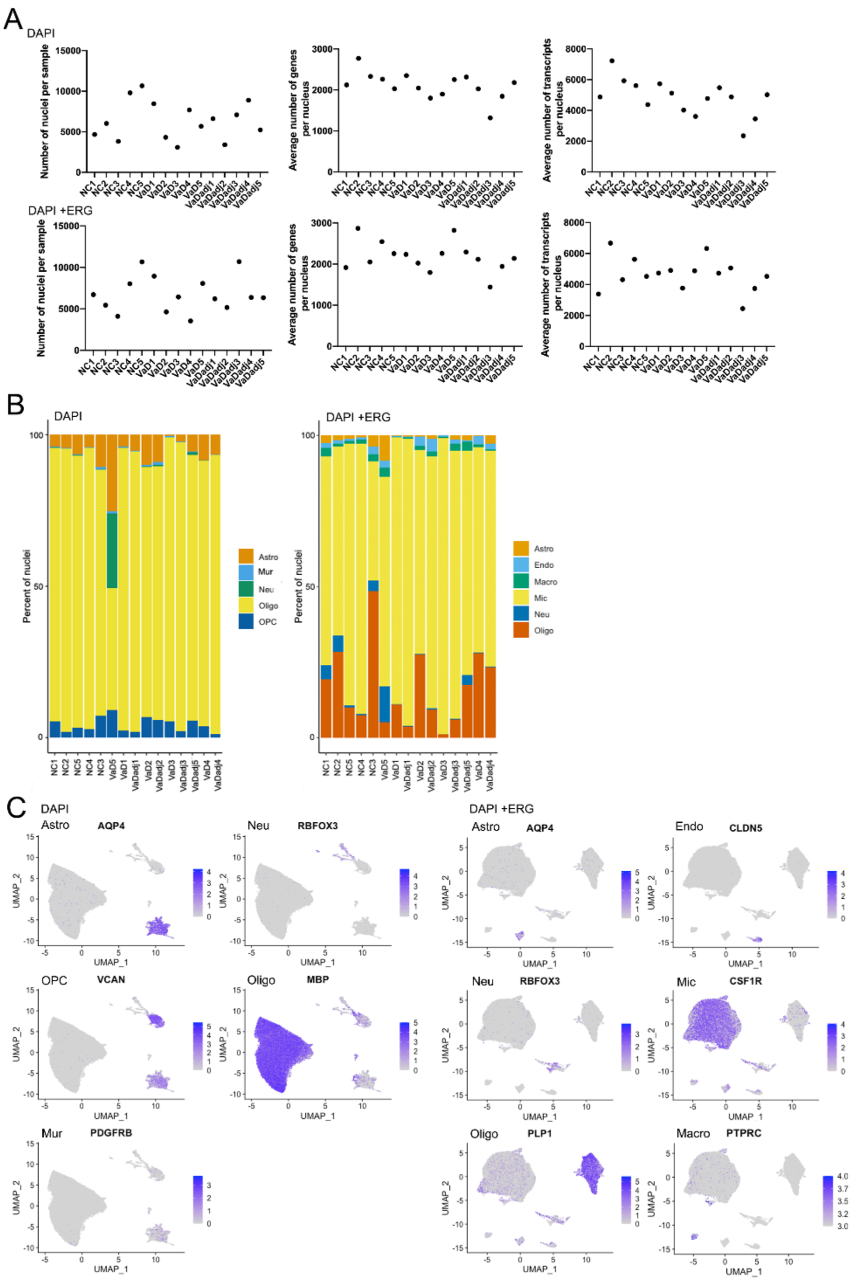

Sup Fig 2. Cell-type heterogeneity in the PVWM (related to Figure 1). A, Number of nuclei per sample, average number of genes and average number of transcripts per nucleus in all 15 samples. B, Proportions of the 5 and 6 major cell types in each sample. C, Expression levels of known markers in each cluster. Astro: astrocytes, Neu: Neurons, OPC: oligodendrocyte progenitor cell, Oligo: oligodendrocyte, Mur: mural cells, Endo: endothelial cells, Mic: microglia, Macro: macrophage.

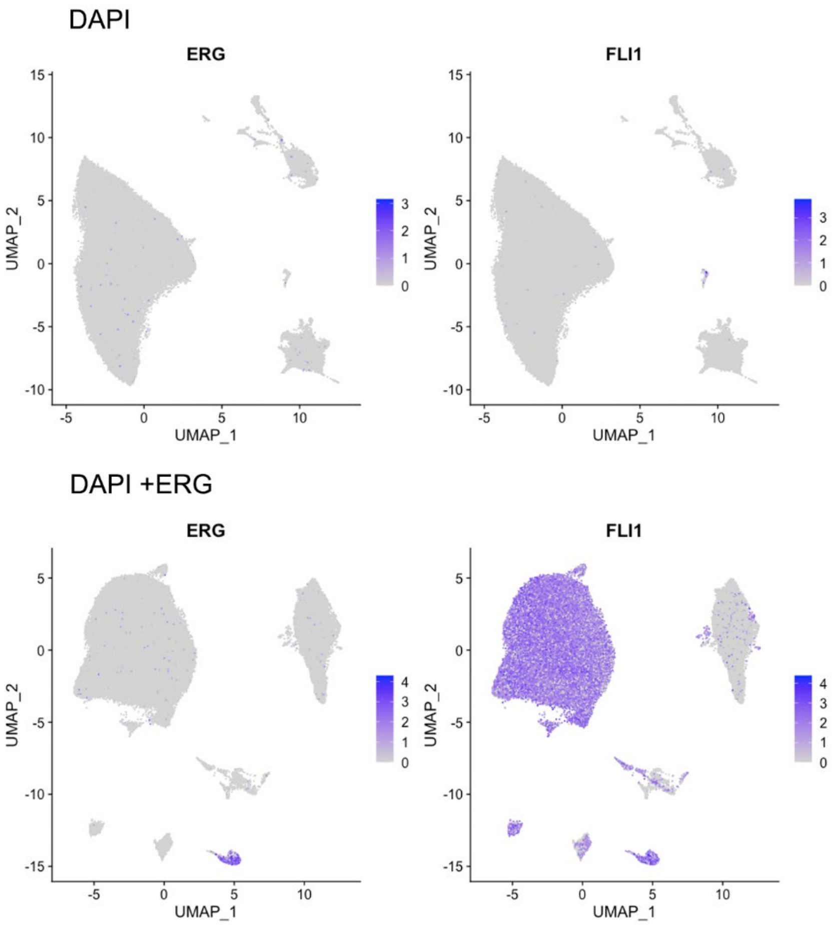

Sup Fig 3. Endothelial cell and microglia enrichment markers (related to Figure 1).

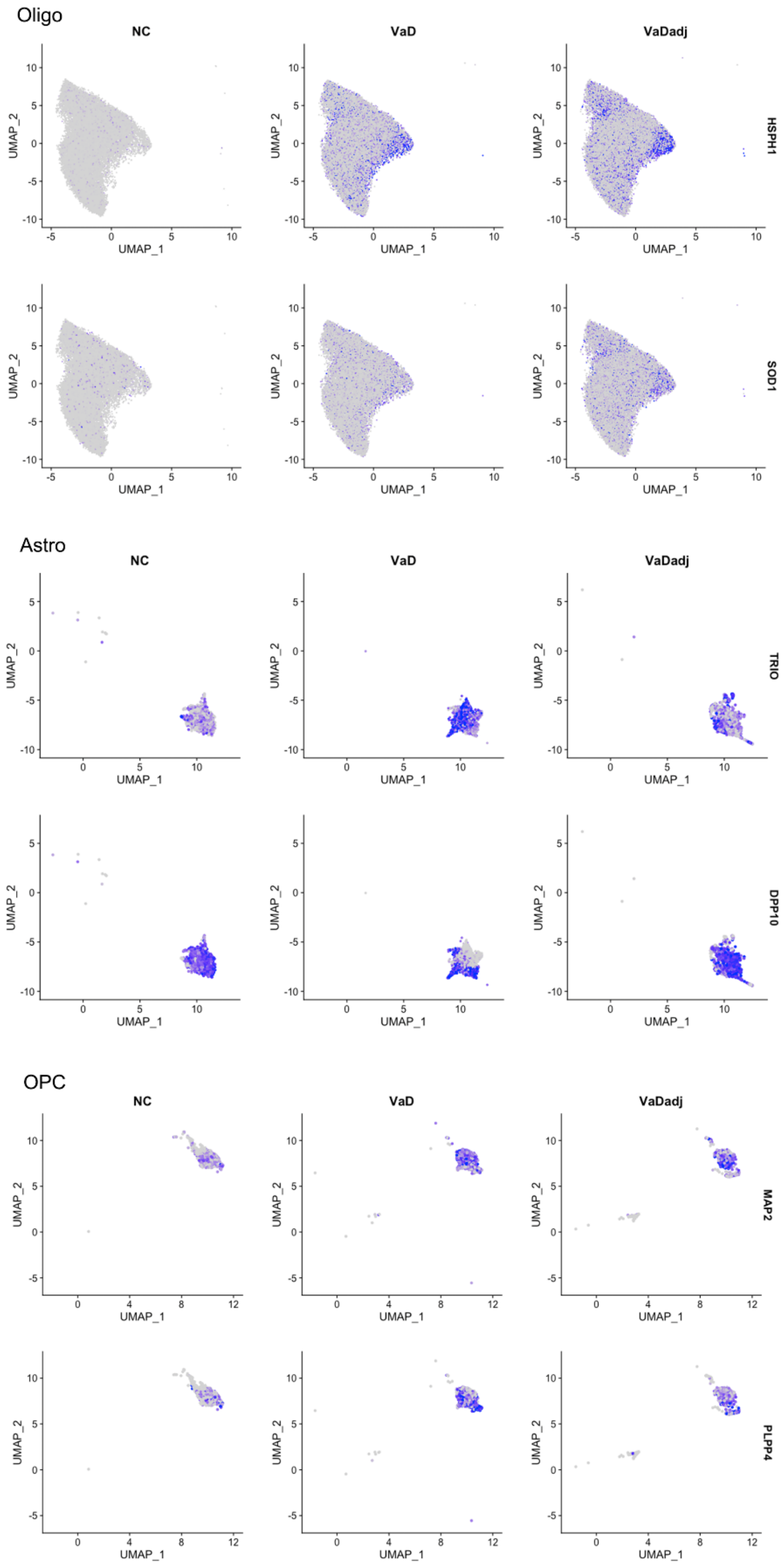

Sup Fig 4. Cell type-specific transcriptomic changes in tissue from patients with Vascular Dementia (related to Figure 2). Projection (UMAP) plots showing the expression levels of the top upregulated genes in each cell type in Vascular Dementia (VaD) and normal control (NC) samples.

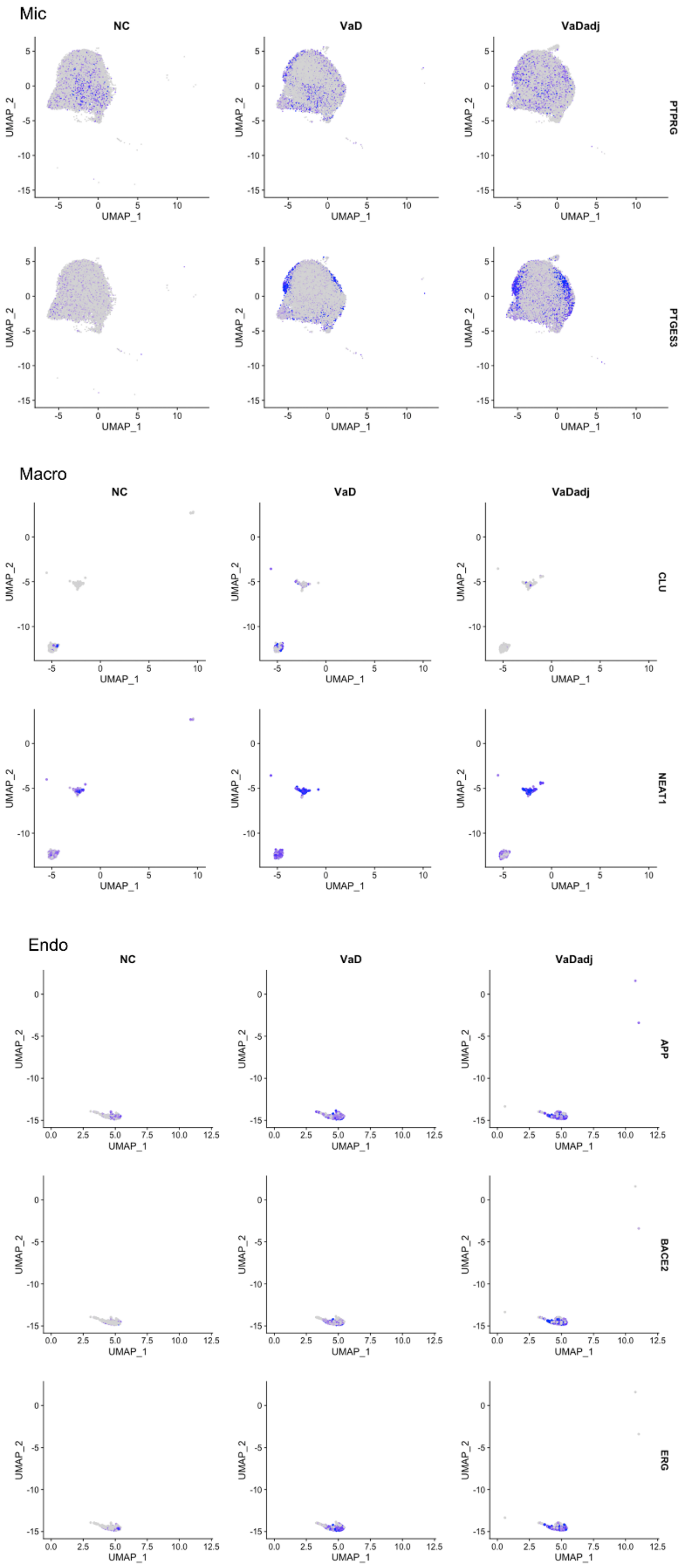

Sup Fig 5. Cell type-specific transcriptomic changes in tissue from patients with Vascular Dementia (related to Figure 2). Projection (UMAP) plots showing the expression levels of the top upregulated genes in each cell type in Vascular Dementia (VaD) and normal control (NC) samples.

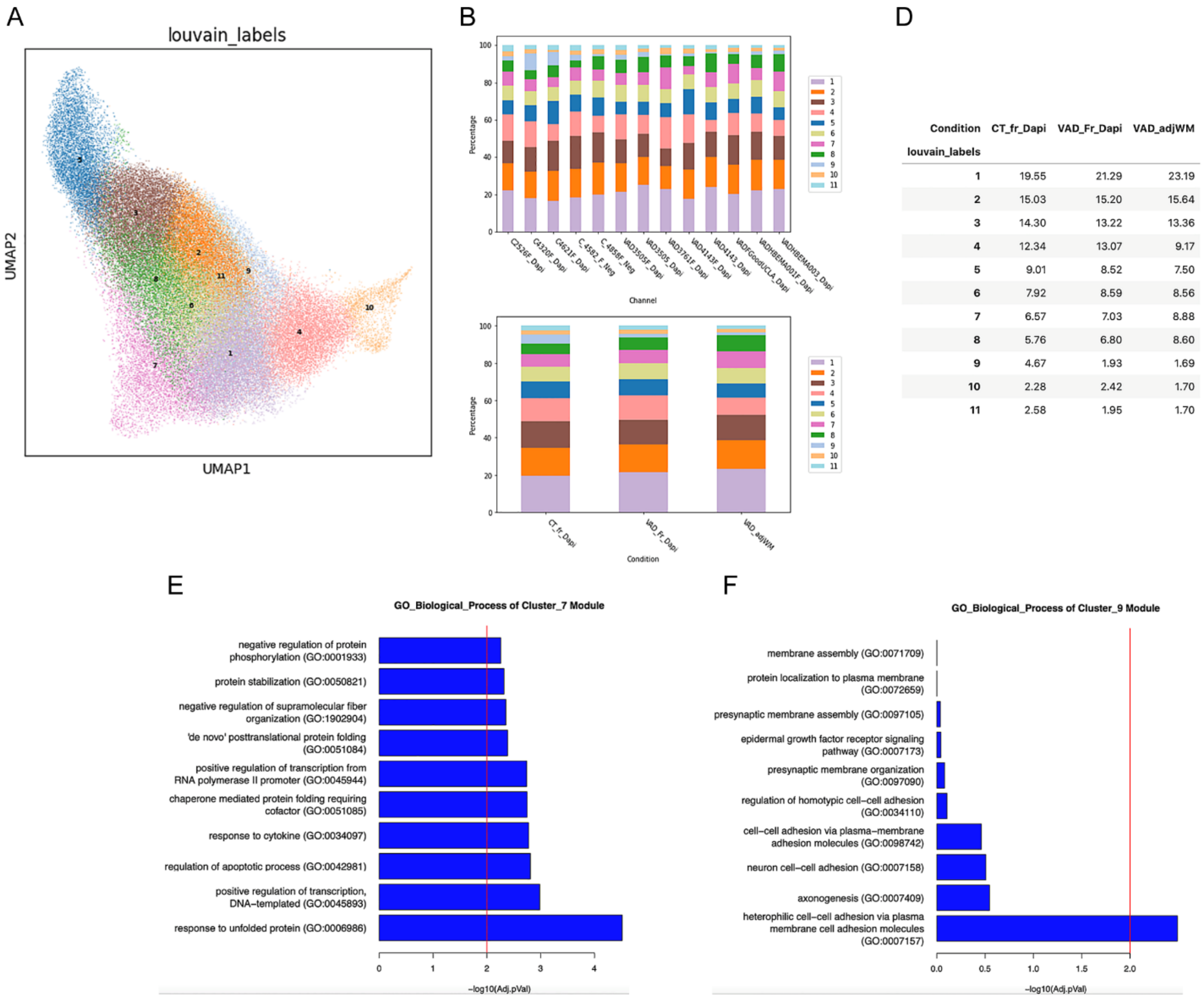

Condition<br>louvain_labels	CT_fr_Dapi	VAD_Fr_Dapi	VAD_adjWM
1	19.55	21.29	23.19
2	15.03	15.20	15.64
3	14.30	13.22	13.36
4	12.34	13.07	9.17
5	9.01	8.52	7.50
6	7.92	8.59	8.56
7	6.57	7.03	8.88
8	5.76	6.80	8.60
9	4.67	1.93	1.69
10	2.28	2.42	1.70
11	2.58	1.95	1.70

Sup Fig 6. Cell type-specific transcriptomic changes in tissue from patients with Vascular Dementia in oligodendrocytes. Conventions as in Figure. 3. A, Projection (UMAP) plot showing subclustering of ODCs based on differentially regulated genes. B, top graph, the percentage of each ODC cluster by sample, with 100% being the total cell for ODCs. C = control (left-most samples), VAD = vascular dementia (middle and right samples). These are cells from the DAPI sample group (not the ERG FACS, which selects for endothelial cells and microglia/macrophages). B, bottom graph, the percentage of total ODC from each subcluster by condition of control (left), VaD lesion (middle) and VaD adjacent (right). D, the percent of total cells of the entire sample that are constituted by each ODC subcluster. CT = control. E,F biological process that to which differentially regulated genes in clusters 7 and 9 belong. The red vertical line indicates statistical significance.

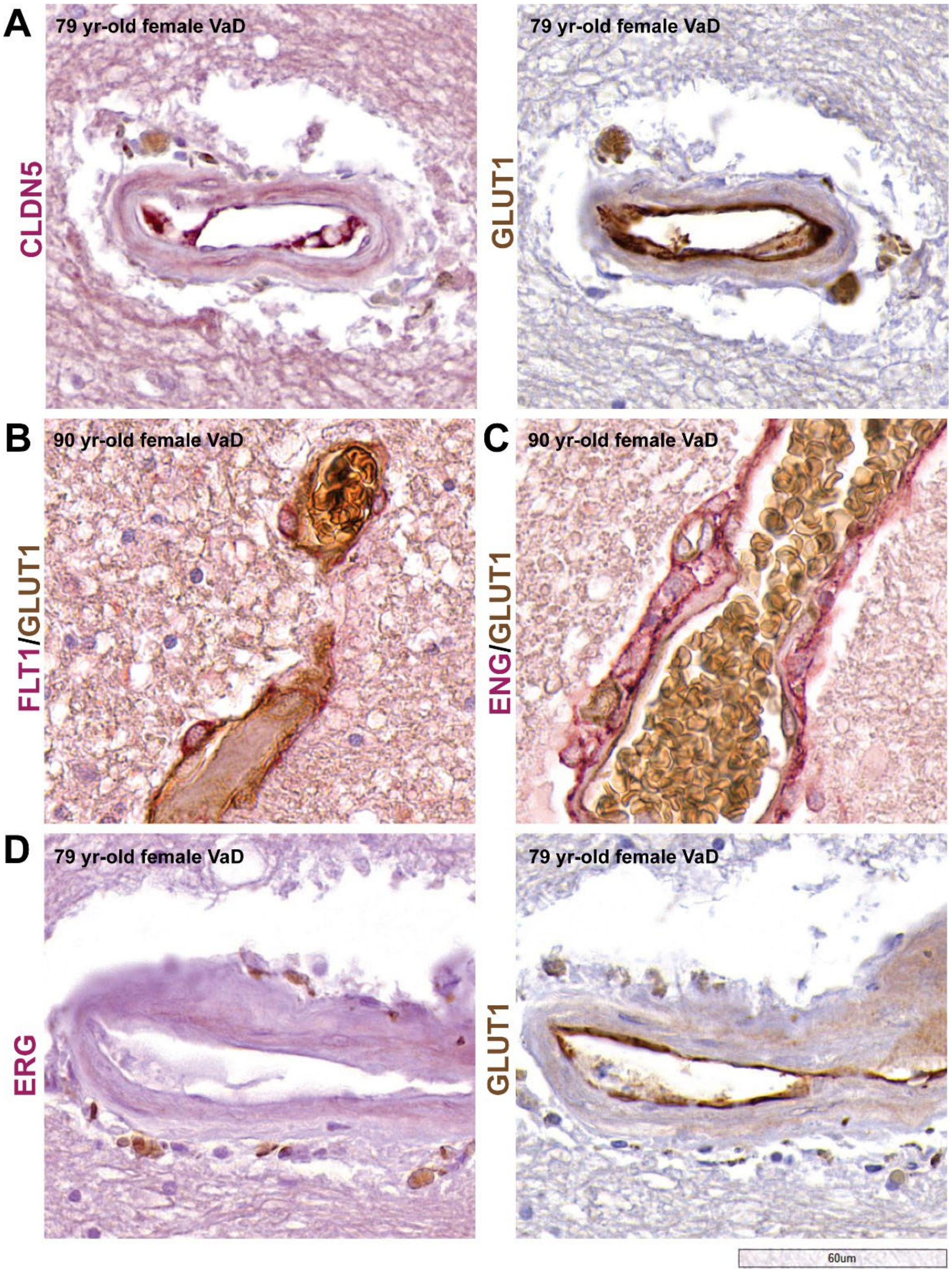

Sup Fig 7. Immunohistochemistry shows the expression of CLDN5, FLT1, ENG, and ERG in PVWM endothelial cells of VaD patients**.** A, Mono color staining of CLDN5 (rabbit antibody) and endothelial cell marker GLUT1 (rabbit antibody) using consecutive brain sections. B, Dual color co-staining of FLT1 (goat antibody) and GLUT1. C, Dual color co-staining of ENG (goat antibody) and GLUT1. D, Mono color staining of ERG (rabbit antibody) and GLUT1 using consecutive brain sections. Scale bar = 60 µm
